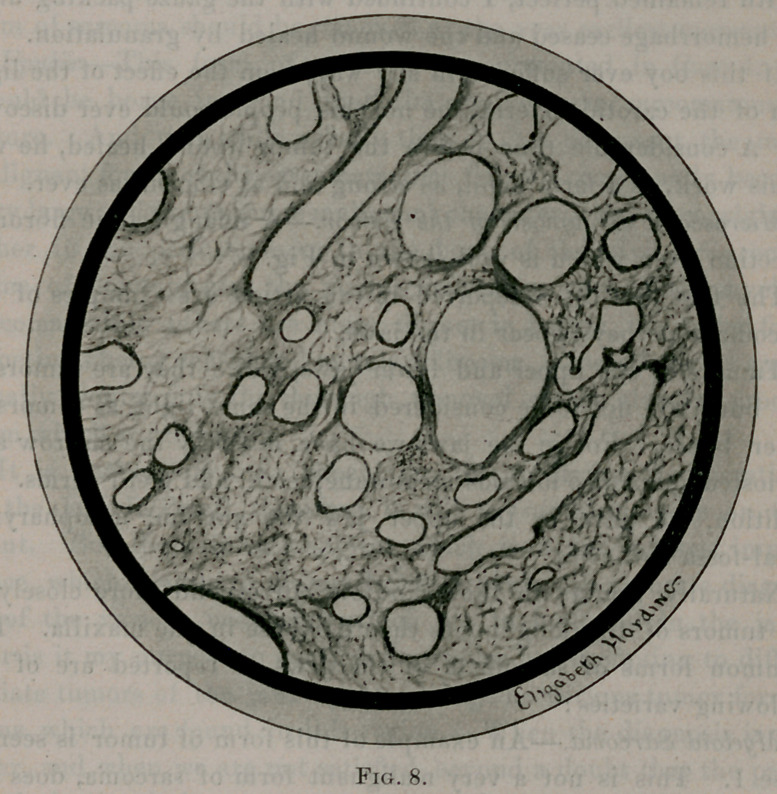# A Report of Some Sarcomata of the Jaws

**Published:** 1899-11

**Authors:** W. H. Hudson

**Affiliations:** Lafayette, Ala.


ATLANTA

### Journal-Record of Medicine.

Successor to Atlanta Medical and Surgical Journal, Established 1855, and Southern Medical Record, Established 1870.

Vol. I. NOVEMBER, 1899. No. 8.

EDITORS { BERNARD WOLFF, M.D., DUNBAR ROY, M.D.

M. B. HUTCHINS, M.D., BUSINESS MANAGER.

PUBLISHED MONTHLY. OFFICE 305 AND 306 FITTEN BUILDING.

### ORIGINAL COMMUNICATIONS.

### A REPORT OF SOME SARCOMATA OF THE JAWS.

By W. H. HUDSON, M.D.,
Lafayette, Ala.

Although it is not my purpose, in this communication, to deal with any other tumors of the jaws, except the sarcomata, a few words concerning malignancy will not be inappproprite. Nor is it my purpose to discuss very deeply the pathology of tumors of the jaws; but to present the cases in their practical bearings, bringing forth only such salient points as will be most useful to the general practitioner, who, after all, is the one who sees these cases first, and really decides for them what should be done.

In my student days I became interested in tumors of the jaws through the influence of my honored preceptor, Dr. Willis F. Westmoreland, Sr., who, as is well known, did some of his most brilliant work along this line.

Malignant tumors of the jaws are comparatively frequent, and it is very important that their true nature be recognized early, for the earlier surgical intervention is given in such cases, the greater are the chances of permanent relief, and the less the deformity. Of course the earlier such operations are done the easier they are to the surgeon. The great surgical principle of *the early and thorough extirpation of malignant disease* should be our guide in these conditions, as in all other conditions of malignancy, and this being the great fact in treatment, it becomes absolutely necessary that we be on the lookout for malignant changes, and bring to bear all means at our command to reach an early and positive diagnosis. There is no subject which comes before the general practitioner which I would stress more than this, and when he holds the welfare of his patients highest in his mind, and I am proud to say that he generally does, he will find out the nature of all tumors wherever they appear, and he will do so at the earliest moment possible. *Whenever and wherever there is an abnormal growth there is the possibility of malignancy, and malignancy must be recognized and gotten rid of without delay.* It should also be borne in mind that benign tumors may become malignant, or they, from their location and size, may demand removal at a later date. So, generally speaking, the sooner benign tumors are removed, the better for both the patient and the surgeon.

In order to avoid making this paper too long, I will report only the following cases, as they represent the more common types of the sarcomata of the jaws. Other cases of tumors of the jaws have been under my care, but would add little to the value of this communication.

CASE No. 1. Operation, August, 1888. Arch P., negro, aged sixteen years. No photograph of this case before operation. Fig. No. 1 shows his condition at the present time, August, 1899.

There was a large tumor of left lower jaw, extending from just below the sigmoid notch to near the mental foramen. The tumor was hard and regular in shape, and on pressure made no crackling sound. The skin was adherent over the extreme external part of the tumor. The teeth could not be separated to the extent of a quarter of an inch, and on this account the boy was brought to me, as the taking of solid food had become very difficult. The lateral movement of the lower jaw was practically destroyed on account of the malar bone being forced upward and outward. The tumor, in the lower jaw, fitting under the malar bone, formed a lock.

FIG. 1.

*Diagnosis.*—Malignancy, perhaps a myeloid sarcoma. The excision of half of the lower jaw advised.

*Operation.*—External incision made from lower lobe of ear to the median line of jaw in front, the portion of skin adherent to the tumor being excised. The lip was severed in the median line; the lateral incisor tooth extracted and the bone cut through with a chain saw. The facial vessels were tied before they were cut, thus avoiding hemorrhage. At this stage of the operation considerable difficulty was experienced on account of not being able to displace the jaw downward or outward. The locking malar bone still effectually holding the tumor fast, most of the muscles had to be cut with the tumor in its original position. Eventually, when all the muscles had been cut, except the temporal, I was enabled to displace the tumor downward and outward. When this movement was accomplished, the inferior dental nerve and artery slipped out of the canal and were very easily handled, the artery tied high up and the nerve cut short. The temporal muscle was easily detached. The wound was closed by deep sutures, and union was complete in about two weeks.

The patient suffered very little shock from the operation, and remained in bed only three or four days. The specimen was lost, and a careful microscopic examination was not made. The tumor was a myeloid sarcoma.

The man from whom this tumor was removed, is now in perfect health—a respected and intelligent school-teacher. He says he suffers very little inconvenience from the loss of half of his lower jaw.

CASE No. 2. Mary G., negro, aged seven years. Fig. 2 shows her appearance before the operation; fig. 3 gives her appearance two weeks afterwards. Operation, June, 1896. This girl had a very large tumor of the lower jaw, extending from the neck of the jaw to the mental foramen. The growth was smooth and solid, and was observed for the first time only three months before she was brought to me; it was, therefore, a very rapid growth. The health of the girl had not suffered to any great extent. There was no history of traumatism.

*Diagnosis.*—A rapidly growing sarcoma; an immediate operation advised, although a not very favorable prognosis was given.

*Operation.*—External excision made as in Case 1. Bone severed near symphysis; hemorrhage was carefully controlled; the tumor was easily displaced downward and outward, and the operation finished with little difficulty. The wound was carefully closed with deeply placed silkworm sutures. The mucous membrane united by catgut. Healing was complete in ten days, and the case discharged. The recovery from the operation was uncomplicated and satisfactory in every respect.

In September, 1896, there were signs of recurrence of the growth. In October a second operation was considered and undertaken. The common carotid artery was tied with the purpose in view of ligating the internal carotid, high up, and then to make a thorough excision of the recurrent growth. After the common carotid was ligated, the little patient exhibited considerable weakness and the operation was abandoned at this point. She suffered in no

FIG. 2.

FIG. 3.

perceptible way from the effect of the ligation of the artery. The sarcoma appeared after this to cease growing so rapidly. A few weeks later the patient passed from my care and died in a short time.

*Examination of Tumor.*—After the tumor was removed it was found to be oblong in shape, perfectly smooth, the bone appearing to grow through its center. Spiculæ of bone were found in all parts of the tumor

*Microscopic Examination.*—A typical spindle-cell sarcoma, ossifying in parts, perhaps originating from the periphery of the bone or the periosteum. There were no giant cells to be found.

The following cases occurred in the practice of Drs. Hudson and Gaines, and I wish here to thank my associate, Dr. W. D. Gaines, for his help and valuable advice. I wish also to thank Drs. W. J. Love and Z. T. Grady for their valuable help in connection with certain of these cases.

CASE No. 3.—C. M., white, aged eighteen years, consulted me in March, 1899, on account of a smooth, conical shaped tumor which grew in front of the first molar, canine, and lateral incisor teeth, on the right side of the lower jaw. The tumor was a little higher than the teeth. The teeth were not displaced backward nor separated. The alveolar process projected outward, showing that the growth extended fully to its lower limit.

FIG. 4.

This tumor is very well shown in Fig. 4. The lateral incisor was found to be very loose and was extracted, bringing away with it a piece of white sarcomatous looking tissue, about the size of half the first phalanx of the little finger. This tissue was strongly adherent to the tooth, and could be cleared from the tooth only by scraping it away with a knife. Microscopic examination showed it to be composed of small round cells and scant stroma.

The patient was informed that the growth was malignant and an early operation advised. She did not, however, consent to an operation until the latter part of April. By this time the growth had considerably increased in size, having extended below the alveolar process, and thickened this process to fully three times its normal thickness.

*Operation.*—Under cocain anesthesia I attempted to saw out the whole disease process, including a small portion of alveolar process on each side of the tumor with a wire saw; but at the beginning of the operation the saw broke and the operation was finished with forceps, chisel and mallet. There was only a very thin shell of bone in front of the tumor, a thicker shell of bone behind. The tumor itself was soft and white, with bony partitions running through it in various directions. The tumor came away from its bed easily, revealing no attachments, leaving the cavity in which it lay perfectly smooth and white. This smooth wall was chiseled away. There was some bleeding from the bone, both in the surface of the cavity, and the alveolar process. This bleeding was effectually stopped by pressing aseptic wax into the bone. The operation was quickly finished, and the patient suffered comparatively little pain. When last seen in August she was well, there being no indication of recurrence.

FIG. 5.

*Microscopic Diagnosis.*—A round-cell sarcoma which, perhaps, developed from the follicle of a tooth, and by pressure had made a large cavity in the bone by displacing it in every direction.

CASE No. 4. J. C., negro, aged twenty-seven. Several years ago noticed a small solid growth on the upper jaw, just at the lower border of the gum, between the canine and first molar teeth on the left side. Two years ago it was removed by a physician, who simply split the tumor and cut out each half. After a few weeks it began to grow again, so that when she consulted me in July, 1899, the tumor presented the appearance as shown in Fig. 5. The patient wished the growth removed, which was easily done, with forceps and chisel, cutting away the alveolar process, from which it evidently had its origin. The time is too short to state whether or not the growth will recur, but in this case I wish more especially to stress the nature of the tumor than the operative principles, as operation for tumors of this kind is very simple.

*Microscopic Diagnosis.*— This tumor was made up of fibers with very few cells, and was what is commonly called a fibrous epulis; but we should bear in mind that the tumor has recurred after it was removed, and the growths that have come under the heading of epulis are sarcomatous in nature, although of a less degree of malignancy than the other forms of sarcomata. I will speak more on this subject shortly.

FIG. 6.

The next case to be reported is not a sarcoma, but on account of the peculiarity of the growth, and the difficulty attending its removal, I report it here:

CASE No. 5. George S., white, aged seventeen years. A healthy farmer lad. Fig. 6 shows his appearance just before the operation. Fig. 7 his appearance four months after the operation. Several years ago it was noticed that the right side of the boy's face, especially that part represented by the malar bone, was wider than the left side. Some time later a small tumor was seen in the right cheek. The growth of the tumor, however, was very slow, and not until the summer of 1898 was any especial attention given it. On examination a firm tumor, about as large as an ordinary size pear, was found occupying the right cheek. The malar bone and the zygomatic arch were displaced outward, making the right side of the face appear very much wider than the left. The tumor was evidently fixed by a large pedicle somewhere under the zygomatic arch.

*Diagnosis.*—It was difficult to reach a conclusion as to the nature of this growth; but it was eventually decided that it was malignant, a sarcoma.

*Operation.*—In the beginning of the operation, two points were especially borne in mind: First, not to injure the duct from the parotid gland. Second, not to cut through the cheek into the cavity of the mouth. An incision three inches long was made over the tumor below and parallel to Stenson's duct. The tumor was easily separated from the surrounding tissue. It did not pulsate and did not indicate, in any way whatsoever, that it was so unusually vascular. It was soon discovered that the pedicle was very large, and that the tumor was very tough. It was also apparent that the pedicle had its origin in the neighborhood of the posterior portion of the upper jaw; perhaps as far back as the wing of the sphenoid bone, and necessarily in close contact with the internal carotid artery and internal jugular vein. The internal maxillary artery and its branches were also near. After the explorative procedure that revealed these facts was finished, there was considerable hemorrhage, and it was my intention to pack the wound with sterile gauze for two days, and then to finish the operation. At that time, however, I caught the pedicle with a large pair of forceps, accidentally tearing it. I then decided to break the pedicle through, pack the wound tightly with sterile gauze, and if the growth proved to be a sarco-

FIG. 7.

ma, which I now began to seriously doubt, being almost positive that it was a fibroma, to do a second operation two days later, removing the point of attachment, as thoroughly as possible. When the pedicle was broken through a furious hemorrhage began, which I endeavored to stop by packing the wound tightly with gauze. The wound was dressed and the patient allowed to come out from under the anesthetic.

I examined the broken pedicle macroscopically and found no evidence of abnormal vascularity. I then decided that the hemorrhage was due to an injury of some of the branches of the internal maxillary artery; but I felt quite assured that the gauze pressure would effectually control it. After a short while, however, the patent's face began to swell, his right eye to bulge; with other indications, all showing very conclusively that a severe hemorrhage was still in progress, I decided, without delay, to ligate the external carotid artery. Within three hours of the first operation the patient was reanesthetized and the common carotid artery exposed at the point where it divides into the internal and external carotids. I now felt a fear that the internal carotid might have been injured, so I placed loose ligatures around both the internal and external carotids. The packing was then removed from the cavity which the tumor occupied, and by the aid of the finger and the ligature, the external carotid artery was closed. The bleeding in the tumor cavity entirely ceased, and after a moment, the ligature around the external carotid was tied, care being taken not to rupture the coats of the artery. The tumor wound was then watched for several minutes, when suddenly a violent hemorrhage began again. The internal carotid was then closed by the finger and ligature, and the hemorrhage promptly ceased. It was fully realized that if the internal carotid was injured that the bleeding could continue by the blood current being reversed from the brain; but as closing the internal carotid stopped the bleeding, and when the internal carotid was opened, the hemorrhage immediately recurred, it was decided to ligate the internal carotid, care being taken not to rupture the arterial coats. After a few moments of watching the tumor cavity a mild hemorrhage occurred, which was easily controlled by gauze packing. The wounds in the neck and cheek were carefully dressed, and after a few hours the boy appeared to be in remarkably good condition. In spite of vigorous remonstrance the boy never kept his bed after the day of the operation.

The ligation wound healed without complication, the stitches being removed on the seventh day. At this time the gauze packing was taken from the tumor wound, and to my surprise another

Fig. 8.

furious hemorrhage occurred, but was readily controlled by repacking.

I now thought it the part of wisdom to examine the tumor which on removal had been placed in Müller's fluid and formaldehyde. The torn portion of the pedicle still revealed no bloodvessels which would account for the bleeding; but when the tumor was incised, to my astonishment, it looked quite like a sponge; pieces cut from the tumor were almost as strong as leather, and could be pressed between the fingers and would expand again, just as a sponge would do. The cause of the bleeding now was very evident. The wound was packed with fresh gauze every week for six weeks, before the hemorrhage ceased. Several times I thought it would be necessary to resort to operative means, to permanently check the bleeding; but as no infection took place, and as the boy's health remained perfect, I continued with the gauze packing until the hemorrhage ceased and the wound healed by granulation.

If this boy ever suffered, in any way, from the effect of the ligation of the carotid arteries, he nor his people could ever discover it. A considerable time before the tumor wound healed, he was at his work, as a farm hand, as strong and as efficient as ever.

*Microscopic Diagnosis of the Tumor.*—A telangiectatic fibroma, a section from which is well shown in Fig. 8.

The first four cases reported in this article are examples of the sarcomata as they appear in the jaws.

Tumors of the upper and lower jaws, while they are tumors of the bone, are not to be considered in the same light as tumors in other bones. For in the jaws we have not only the marrow and periosteum, but also mucous membrane, teeth, and teeth germs. In addition, we have in the upper jaw the antrum, nasopharynx, nasal-fossa and orbit.

Naturally, tumors in the mandible correspond more closely to the tumors of the long bones than do those in the maxilla. The common forms of sarcomata of the jaws as reported are of the following varieties:

*Myeloid Sarcoma.*—An example of this form of tumor is seen in Case 1. This is not a very malignant form of sarcoma, does not early infiltrate the surrounding tissue, and is therefore favorable for operation, especially when the operation is done early.

*Periosteal Sarcoma.*—Of this form there are two varieties—the spindle-cell and the round-cell. Case 2 represents the spindle-cell variety. This is a very malignant form of sarcoma, and operations done for its removal should be done very early and thoroughly.

*Sarcoma Having its Origin in a Tooth Follicle.*—Case 3 is an example of this form of sarcoma. Sarcomata of this variety have been mistaken for the myeloid form, and on this account, perhaps, some writers have considered the myeloid form of sarcoma as of frequent occurrence. These sarcomata, originating in tooth follicles, are at first encapsulated, but later infiltrate the surrounding structures, producing regional infection. It has been claimed this form of sarcoma only appears in children, but the case reported here shows that it may form in a person eighteen years old. This form of sarcoma should be removed at the very earliest moment.

*Epulis.*—This form of sarcoma is represented in Case 4. It should be borne in mind that the epulides are sarcomatous in nature. And that while usually they do not represent the more malignant forms of the sarcomata, that they do occasionally become very large and even assume malignant characteristics; they originate either in the periosteum or in the bone of the alveolar process. Some of them present the microscopic appearance of the myeloid sarcoma, while others are more fibrous in nature, the latter form sometimes being considered merely a fibroma. Operations for epulis usually require only the thorough removal of the alveolar process from which it originates.

It is unnecessary in a paper like this to discuss the operations on the jaws in detail, therefore I will say nothing more on this point. But there is one subject which is of the greatest importance, which I wish to discuss briefly, that is, the accurate diagnosis of the various neoplasms which are to be found in the jaws. Nor is it my purpose to confuse the reader by attempting to differentiate tumors of the jaws by describing the various tumor formations which are found in this region. When the diagnosis is not clear, and when we are not satisfied beyond a doubt that the pathological growth is not a cyst, nor an odontoma, nor fibroma, nor any other benign form of tumor, it is our duty, if necessary, to make an exploration into the tumor and satisfy ourselves by microscopic examination as to its nature before a mutilating operation is performed.

All advance which we can expect to make along the line of treatment in malignant diseases is by early and accurate diagnosis, and early and thorough removal of the malignant formation.

## Figures and Tables

**Fig. 1. f1:**
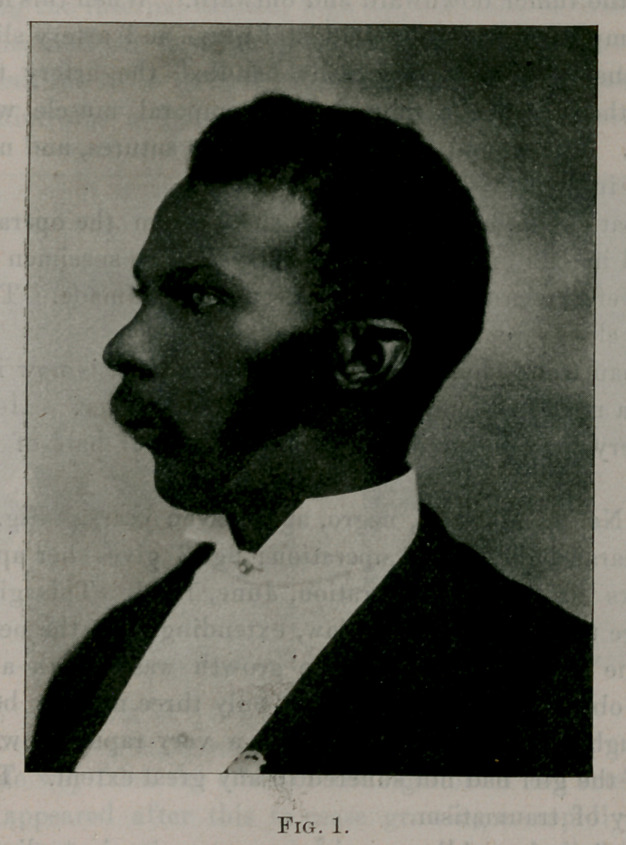


**Fig. 2. f2:**
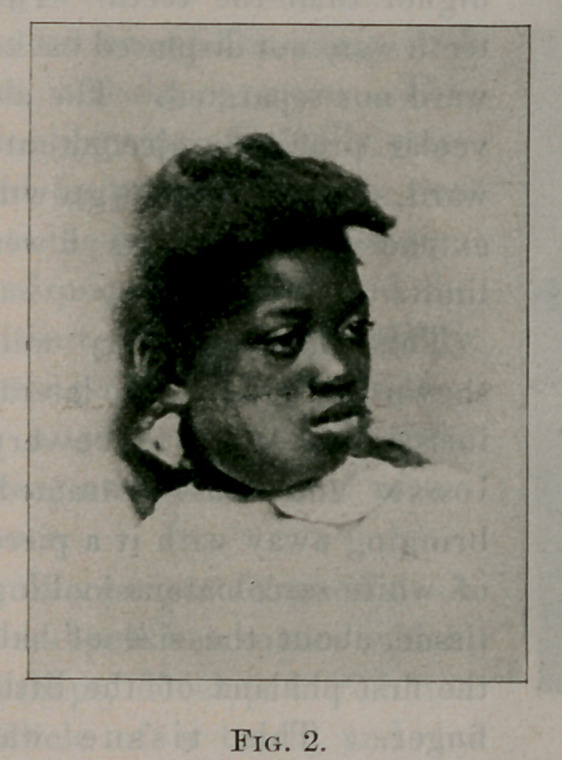


**Fig. 3. f3:**
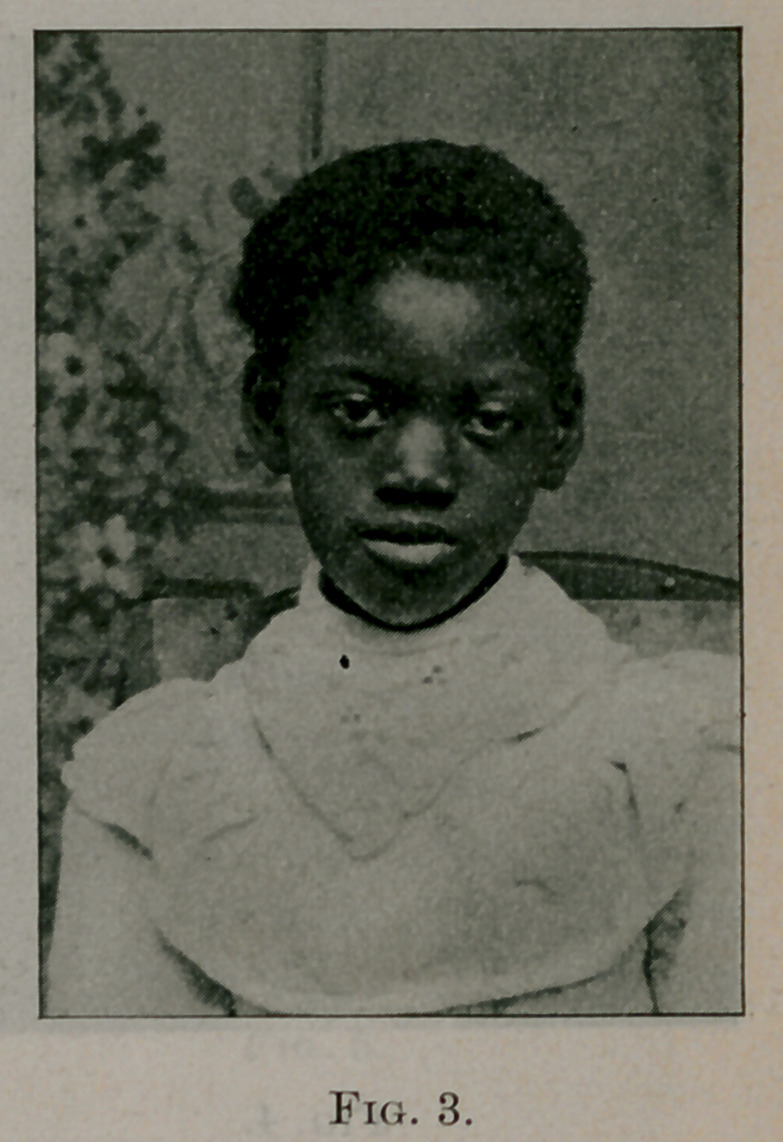


**Fig. 4. f4:**
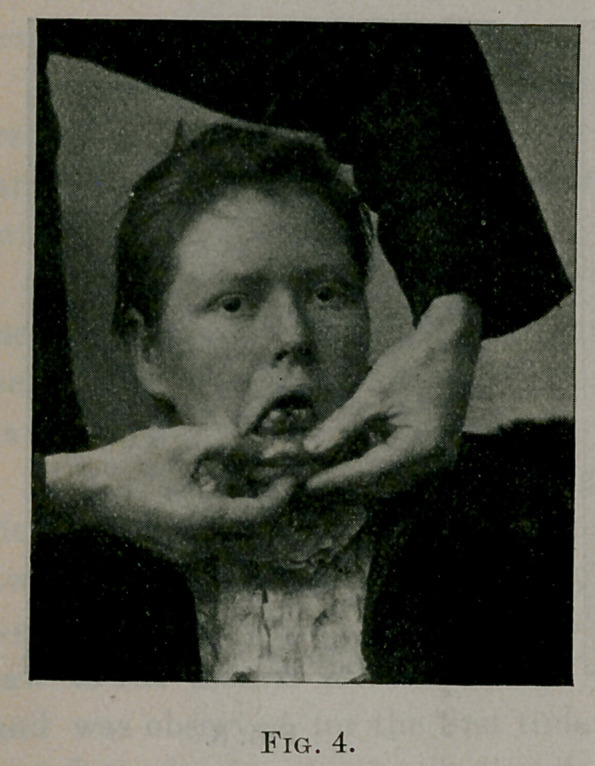


**Fig. 5. f5:**
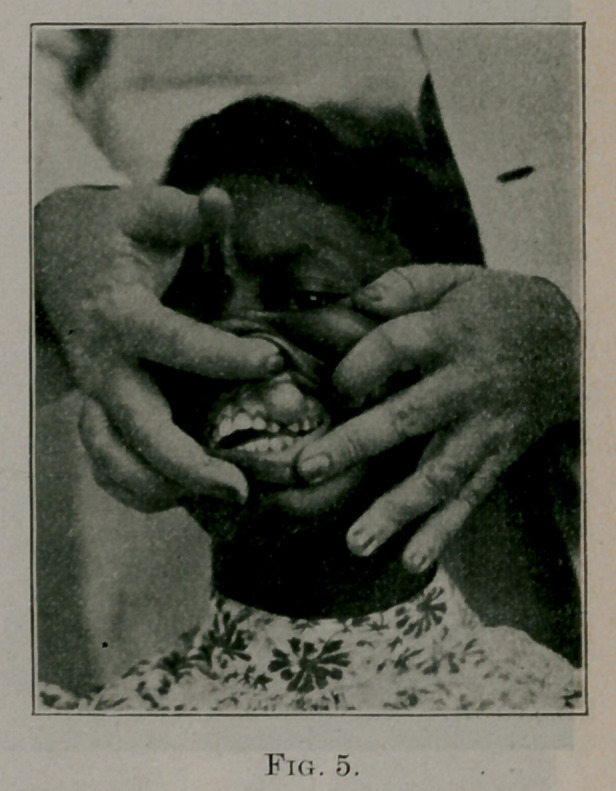


**Fig. 6. f6:**
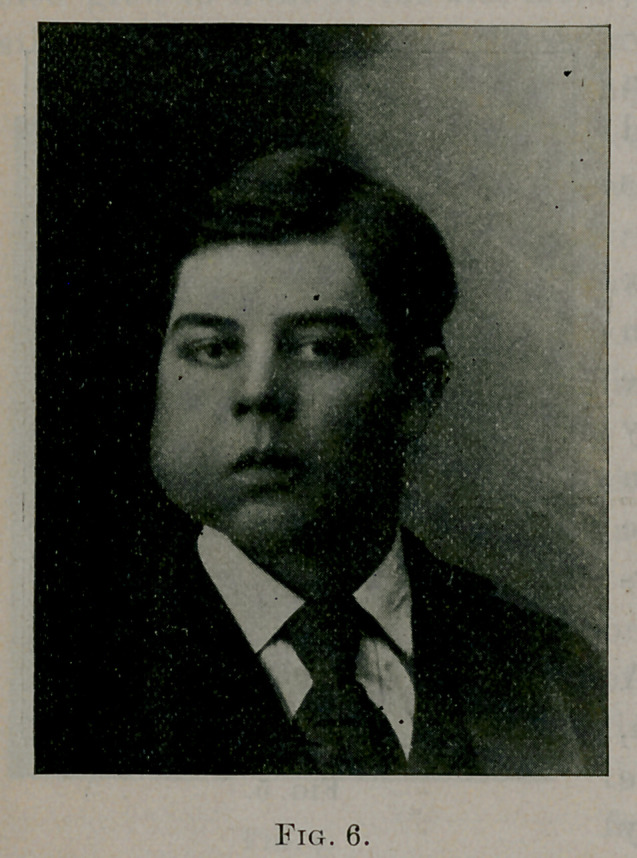


**Fig. 7. f7:**
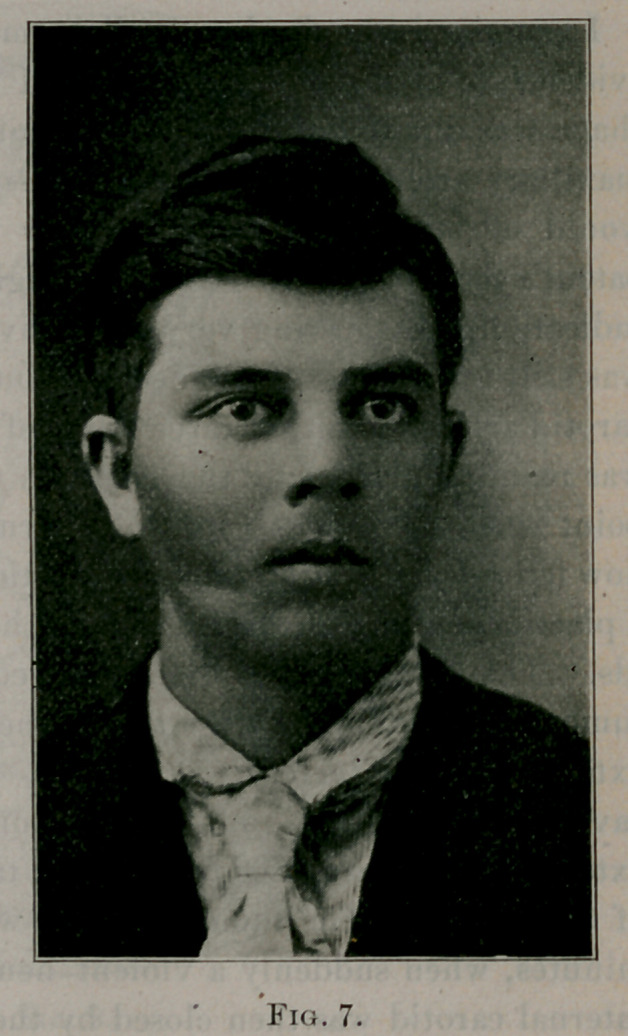


**Fig. 8. f8:**